# Individual, health system, and contextual barriers and facilitators for the implementation of clinical practice guidelines: a systematic metareview

**DOI:** 10.1186/s12961-020-00588-8

**Published:** 2020-06-29

**Authors:** Verónica Ciro Correa, Luz Helena Lugo-Agudelo, Daniel Camilo Aguirre-Acevedo, Jesús Alberto Plata Contreras, Ana María Posada Borrero, Daniel F. Patiño-Lugo, Dolly Andrea Castaño Valencia

**Affiliations:** Facultad de Medicina, Universidad de Antioquia, Grupo de Investigación Rehabilitación en Salud, Carrera 51 D # 62-29 oficina MUA 302, Medellín, Colombia

**Keywords:** Evidence-based practice, Implementation science, Clinical practice guidelines, Health services research

## Abstract

**Introduction:**

Clinical practice guidelines (CPGs) are designed to improve the quality of care and reduce unjustified individual variation in clinical practice. Knowledge of the barriers and facilitators that influence the implementation of the CPG recommendations is the first step in creating strategies to improve health outcomes. The present systematic meta-review sought to explore the barriers and facilitators for the implementation of CPGs.

**Methods:**

A search was conducted in the PubMed, Embase, Cochrane, Health System Evidence and International Guideline Library (G-I-N) databases. Systematic reviews of qualitative, quantitative or mixed-methods studies that identified barriers or facilitators for the implementation of CPGs were included. The selection of the title and abstract, the evaluation of the full text, extraction of the data and the quality assessment were carried out by two independent reviewers. To summarise the evidence, we grouped the barriers and facilitators according to the following contexts: political and social, health organisational system, guidelines, health professionals and patients.

**Results:**

Overall, 25 systematic reviews were selected. The relevant barriers in the social-political context were the absence of a leader, difficulties with teamwork and a lack of agreement with colleagues. Relevant barriers in the health system were a lack of time, financial problems and a lack of specialised personnel. Barriers of the CPGs included a lack of clarity and a lack of credibility in the evidence. Regarding the health professional, a lack of knowledge about the CPG and confidence in oneself were relevant. Regarding patients, a negative attitude towards implementation, a lack of knowledge about the CPG and sociocultural beliefs played a role. Some of the most frequent facilitators were consistent leadership, commitment of the members of the team, administrative support of the institution, existence of multidisciplinary teams, application of technology to improve the practice and education regarding the guidelines.

**Conclusions:**

The barriers and facilitators described in this review are factors that influence the implementation of evidence in clinical practice. Knowledge of these factors should contribute to the development of a theoretical basis for the creation of CPG implementation strategies to improve professional practice and health outcomes for patients.

## Background

Evidence-based clinical practice guidelines (CPGs) are intended to assist practitioner and patient decisions about appropriate healthcare for specific clinical circumstances. However, studies in countries such as the United States and the Netherlands have suggested that at least 30–40% of patients do not receive care according to current scientific evidence [1]. Translating evidence from CPGs into practice, also known as implementation [2], is a challenging process as it involves making changes at the individual, organisational or health system levels. Identifying the factors that influence the implementation of the recommendations, that is, the barriers and facilitators of the process, is an important first step to achieve those changes and bring effective interventions into practice [3, 4].

In previous evidence synthesis studies, different factors have been described that can influence the clinical decisions of health professionals. In 2008, a systematic meta-review with a search strategy conducted in 2006 concluded that the factors that influence the implementation of CPGs could be classified in those related to the characteristics of the guidelines, implementation strategies, professionals, patients and the environment [5]. Later, in 2013, Flottorp et al. [6] developed a systematic review, a synthesis of frameworks and an expert consensus process to develop a checklist of factors that prevent or enable improvements on healthcare professionals’ practice. They found 57 factors and grouped them in those related to guidelines, individual health professionals, patients, professional interactions, incentives and resources, the capacity for organisational change, and social, political and legal factors.

The increasing literature in this area, in the form of systematic reviews and research synthesis, may overwhelm healthcare professionals, managers and other decision-makers seeking to understand how best to implement CPGs. A meta-review of systematic reviews can provide a broad synthesis of the existing evidence in a single manuscript that might be helpful to inform practice, research and policy [7].

The objective of the current study was to carry out a systematic meta-review of reviews that explore the barriers and facilitators for the implementation of CPGs in the different clinical areas of health and according to the socio-political contexts, the health organisational system, the CPG itself, the individual and the patient.

## Methods

### Study design

We conducted a meta-review, namely a systematic literature study of existing relevant systematic reviews. The structure of the review process was adapted according to the guidelines of the Cochrane Collaboration [8] and the reporting follows the Preferred Reporting Items for Systematic Reviews and Meta-Analyses (PRISMA) guidelines [9].

### Review protocol and registration

We developed but did not publish or register a protocol for this meta-review of barriers and facilitators for the implementation of clinical practice guidelines. Meta-reviews cannot be registered with the International Prospective Register of Ongoing Systematic Reviews (PROSPERO). The protocol can be found in Additional file 1.

### Selection criteria

We included systematic reviews or meta-analyses, or other types of systematic evidence synthesis, of qualitative, quantitative or mixed-methods primary studies that identified barriers and facilitators for the implementation of CPGs, from any area of health, whose target populations were patients and providers of health services and that were published in the period from December 2006 to January 2018 and in any language.

We excluded narrative reviews, primary studies, studies of implementation tools or adherence to treatments.

### Search strategy

The meta-review published by Francke et al. [5] included studies that were published until November 2006; therefore, a search was conducted from December 2006 to February 2017 and updated between February 2017 until 10 January 2018. We searched PubMed, Embase, Cochrane, International Guideline Library (G-I-N) and Health System Evidence databases with the terms “Guidelines”, “barriers” and “implement*”, with their respective medical subject heading (MeSH) and synonyms, applying the subset to select systematic reviews, meta-analyses and guidelines (Additional file 2). A manual search according to the references of the articles found was also performed using the snowball methodology.

### Identification of studies and data extraction

After eliminating the duplicates, two independent researchers (VC and AC) reviewed the titles and abstracts according to the established selection criteria, and disagreements were resolved by a third evaluator (JP). Next, a full-text review was conducted by two independent reviewers (JP and VC), and disagreements were resolved by a third evaluator (LL).

The extraction of data was carried out using forms designed by the researchers with the following information: the country where the included individual studies in each review were conducted, level of income of those countries, level of care (i.e. primary, secondary or tertiary care), health theme, type of review (quantitative, qualitative or mixed according to the type of studies that were included in the review) and the results and conclusions as synthesised by the review authors.

### Quality evaluation

The quality evaluation of the reviews was conducted independently by two reviewers with the tool developed by the Joanna Briggs Institute (JBI) “Checklist for Systematic Reviews and Research Syntheses” [10]. This tool can be used in systematic meta-reviews reviews in which quantitative or qualitative systematic reviews can be incorporated. The checklist consists of 11 questions, each of which must be answered as “yes”, “no”, “uncertain” or not applicable “NA”. For each review, we provide an average score of the two reviewers (Additional file 3).

### Summary of the evidence

**Fig. 1:**
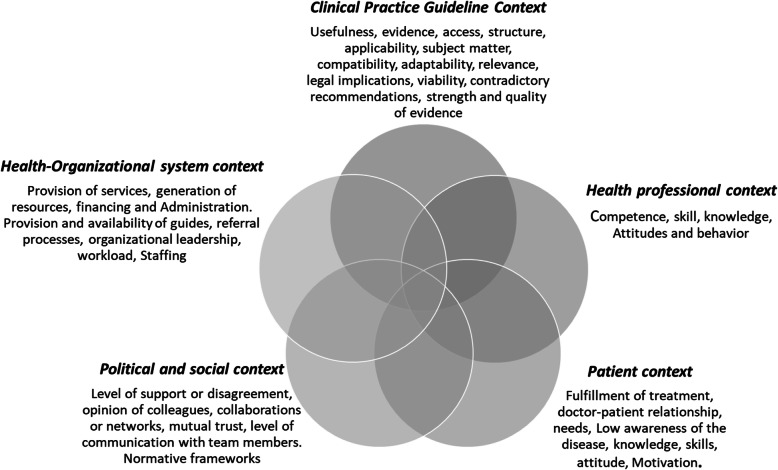
Contexts to explore barriers and facilitators for CPG implementation

The synthesis of the evidence was carried out in different stages. First, after several discussions within the research group, informed by the findings of systematic synthesis [5, 6], we created five contexts to group the barriers and facilitators; these contexts are the political and social, the health organisational system, the guideline, the health professional and the patient context. Figure 1 describes some of the characteristics of each context.

Then, the barriers and facilitators described in the results and discussion sections of the included reviews were extracted and classified under the contexts mentioned in the first stage. In the third stage, a matrix was created with the reviews in the columns and the contexts in rows, which allowed us to compare the extracted descriptions across the included reviews within each context; this process permitted the identification of frequent and recurrent barriers and facilitators in the literature. For each context, we list the barriers and facilitators from the most to the least frequently mentioned. In this stage, we also selected text quotes that could reinforce the meaning of the identified barrier of facilitator. Finally, we built a general interpretation for each context according to the findings in the previous stage. This method of synthesis is similar to the meta-ethnography method described by Dixon-Woods et al. [11].

## Results

**Fig. 2:**
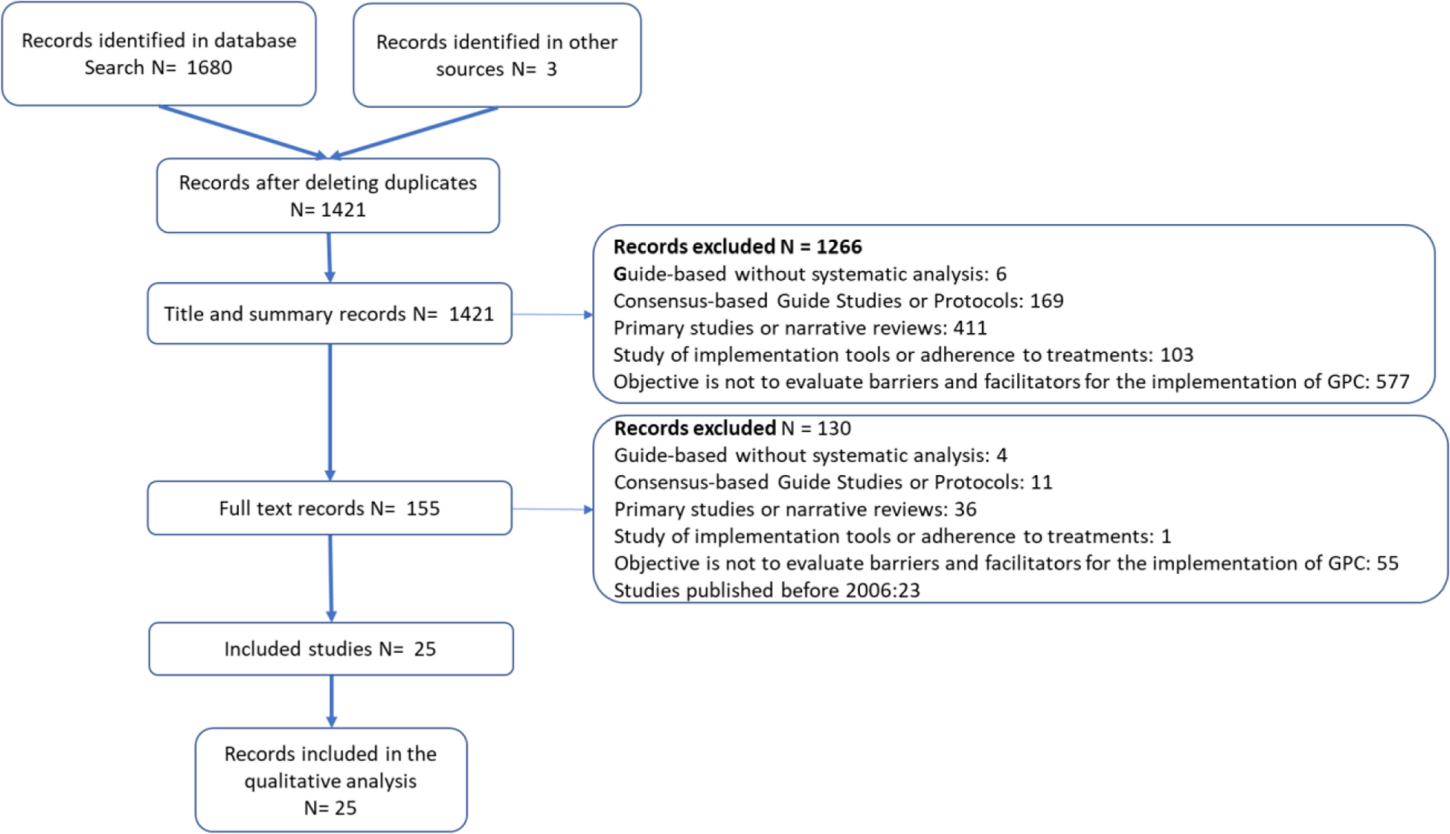
Flowchart of search and exclusion process

After eliminating duplicates, 1420 titles were obtained. A total of 154 articles were selected for full-text review and 25 reviews were selected for inclusion. Inter-rater reliability was assessed via the kappa statistic and was 0.54. Disagreements were resolved by consensus. The causes for which the studies were excluded from this meta-review are recorded in the flowchart [9] (Fig. 2).

### Included studies

The reviews included 960 primary studies that were conducted in different countries, including the United States, the United Kingdom, Australia, Canada, the Netherlands, Germany, Switzerland, Israel, Ireland, Sweden, and various African countries. Two reviews [12, 13] included low-income countries, namely Tanzania, Nigeria, India, Ethiopia, Ghana, Kenya, Uganda, Pakistan, Kosovo and Malawi. Seven reviews involved primary care [14–20], two systematic reviews covered primary, secondary and tertiary care [12, 17], and 15 did not specify the level of care. A total of 87.5% of reviews used quality assessment tools for the included studies, and within these, the Critical Appraisal Skills Programme was used in 38%. Only two reviews had a quality score of less than 6 out of 11 criteria and were included given their relevance to our topic. The characteristics of the studies and the quality of the evidence are shown in Table 1.

**Table 1:** Characteristics of included studies

Review	Countries where the primary studies were conducted	Health theme	Type of review		Quality JBI
Slade et al. (2015) [21]	Canada, United States, Netherlands, Israel, New Zealand, Germany, United Kingdom and Norway	Low back pain	SR	Qualitative	8/10
Baatiema et al. (2017) [18]	Australia, Usa, Switzerland, Denmark, Netherlands, Norway	Cerebrovascular disease	SR	Mixed	9/11
Chan et al. (2017) [22]	United States	Dyslipidaemia, high blood pressure and overweight/obesity	RR	Qualitative	8/10
Craig et al. (2016) [23]	United States, France, Australia, Sweden, The Netherlands	Cerebrovascular disease	SR	Mixed	9/11
Egerton et al. (2017) [24]	Australia, France, United Kingdom, Germany and Mexico	Osteoarthritis	SR	Qualitative	8/10
Eisner et al. (2011) [25]	Switzerland	Infectious diseases and prevention activities	SR	Mixed	9/11
Gaston et al. (2011) [26]	United States, Australia, Saudi Arabia, United Kingdom, Iran, Ireland, Canada	Venous thromboembolism	SR	Mixed	10/11
Ince et al. (2016) [27]	United Kingdom	Schizophrenia, cognitive behavioural therapy and family intervention	SR	Mixed	9/11
Jun et al. (2016) [19]	United States, Australia, Canada, Finland, Singapore, Sweden and the Netherlands	Nursing Clinical Practice Guidelines	SR	Mixed	8/11
Stokes et al. (2016) [12]	Sub-Saharan Africa, Somalia, Tanzania, Burkina Faso, Benin, Senegal, South Africa	Obstetric care	SR	Qualitative	9/10
Rushforth et al. (2016) [28]	United States, United Kingdom, Asia, Africa, Europe (not United Kingdom)	Diabetes mellitus 2	SR	Qualitative	8/10
Rubio-Valera et al. (2014) [14]	United Kingdom, Denmark, United States, Sweden, Switzerland, Spain, Germany, Israel, Ireland, Netherlands, Canada, Australia, New Zealand	Chronic diseases; promotion and prevention of health in primary care	SR	Qualitative	9/10
Khatib et al. (2014) [29]	United States, Canada, United Kingdom, Israel, Brazil, Korea, Australia, Netherlands, India, Egypt, Switzerland, Ireland, Trinidad, Croatia, China, Russia, Nigeria, Malaysia, South Africa, Kuwait, Singapore	Arterial hypertension	SR	Mixed	10/11
De Vleminck et al. (2013) [30]	United States, Canada, United Kingdom, Netherlands, Australia, Singapore, Belgium, Israel	Advance care planning	SR	Mixed	9/11
Siabani et al. (2013) [31]	Sweden, United States, United Kingdom, New Zealand, Canada, Australia, Malaysia	Chronic heart failure	SR	Qualitative	7/10
Sadeghi-Bazargani et al. (2014) [17]	United Kingdom, United States, Netherlands, India, Canada, Australia, Poland, Finland, Jordan, Belgium, Africa, Chile, Argentina, China, Japan, Ireland, Malaysia, Saudi Arabia, Iran, Switzerland, South Korea, Germany	EBM in primary healthcare, secondary and specialised care	RR	Mixed	10/11
Wood et al. (2017) [15]	United States, United Kingdom, Germany, Canada	Depression (collaborative attention)	SR	Qualitative	10/10
Gravel et al. (2006) [32]	Canada, United Kingdom, United States, Netherlands, Australia, France, Mexico, Norway, Germany, China	Shared decision-making	SR	Mixed	9/11
Busetto et al. (2015) [20]	United States, Belgium, Austria, Israel, Canada, United Kingdom, Germany	Diabetes	SR	Mixed	10/11
Lau et al. (2015) [16]	United States of America, Canada, the United Kingdom, Australia and Europe	EBM	RR	Mixed	8/11
Cochrane et al. (2007) [33]	No data	EBM	SR	Mixed	7/11
Samnani et al. (2017) et al. [13]	Bangladesh, Afghanistan, Ethiopia, Ghana, Kenya, Uganda, Northern Nigeria, Tanzania, India, Pakistan, Kosovo, Malawi, Myanmar, sub-Saharan African countries (Democratic Republic of the Congo, Maban and Burkina Faso)	Obstetric haemorrhage	SR	Mixed	7/11
Christl et al. (2011) [34]	Australia	Prevention of cardiovascular diseases	SR	Mixed	4/11
De Clercq et al. (2017) [35]	United States, United Kingdom, Canada, Australia, Italy, Germany, Switzerland, Israel and Poland	Paediatric palliative care	SR	Mixed	5/11
Flottorp et al. (2013) [6]	No data	Healthcare setting and public health services and clinical services	SR	Mixed	8/11

*EBM* Evidence-based medicine, *RR* Review of revisions, *SR* Systematic review

### Synthesis of barriers and facilitators for CPG implementation

In this section, we present the most frequent barriers and facilitators according to each context in the text and other no so frequent but relevant barriers and facilitators (Tables 2 and 3).

**Table 2:** Other barriers for the implementation of Clinical Practice Guidelines

Contexts	Other barriers
Political and social context	• Difficulties in prioritising the health problem [16, 20, 29] • Lack of access to information, lack of mechanisms and systems to support storing of information [6, 13, 16, 20]
Health organisational system context	• Lack of protocols and processes that clearly define the roles within the institution to implement guidelines [19, 28, 29, 34, 35] • Additional workload [6, 14, 16, 18–20, 27, 28, 34] • Difficulty accessing health services [18, 21, 28, 29, 32] • Difficulties with availability of medicines [13, 18, 24, 27] • Deficiency in staff continuous education [18, 20, 27, 28] • Deficiencies in the referral of patients to services [18, 23, 27] • Lack of skill and specialist knowledge within services • Insufficient support from institutions [15, 18, 30] • High turnover of staff that prevents a continuous training process [18, 20, 23] • Limitations of infrastructure [18, 20, 29] • Lack of availability of interpreters in services [20, 28, 29] • Lack of access to information, lack of mechanisms and systems to support storing of information [13, 16, 20]
Guidelines context	• Lack of awareness of the existence of guidelines and clarity of guidelines [13, 16–19, 23, 28] • Beliefs that the guidelines evidence is incorrect or not enough to be reported [17, 19, 27, 35] • Beliefs that CPG is too rigid, may not always be practical and cannot be applied on a day-to-day [16, 19, 29, 32] • Guidelines restrict clinical judgment and challenge professional autonomy and limits treatment options [18, 20]
Health professional context	• Greater confidence in clinical experience than in guidelines recommendations [17, 19, 20, 24, 27, 31] • Lack of effective communication, research and self-learning skills [16, 17, 20, 23, 25, 28–30] • Resistance to change caused by disagreement with the recommendations of the CPG, doubts about the efficacy of interventions and clinical outcomes [16, 18, 22, 24, 29, 32] • Physician’s reluctance to use CPG because of patient factors, self-belief or fear of complications [16, 18, 20, 23, 26, 29] • Little familiarity with guideline recommendations [18, 23, 28, 29, 32] • Negative attitudes of physicians towards the implementation of the guideline or to EBM [16, 17, 19, 24, 27] • Lack of autonomy and authority [17, 18] • Belief that intervention was not part of their role [16, 26, 30]
Patient context	• Language and literacy problems [18, 20, 24] • Lack of motivation, compliance and knowledge to follow the recommendations [20, 25, 28, 29] • Patient comorbidities, mobility problems, polypharmacy and self-empowerment capacity [20, 26, 28, 29] • Patients’ financial situation and occupational status [20] • Depression, anxiety and fear [28–31]

*CPGs* clinical practice guidelines

**Table 3:** Other facilitators for the implementation of Clinical Practice Guidelines

Contexts	Other Facilitators
Political and social context	• Appropriate use of technology and integrated information systems [15, 19] • Clear communication between professionals and management, with defined roles and responsibilities [16, 19] • Positive working relationships between health workers [12, 16] • Financial incentives to achieve some positive goals for the implementation [14] • Adequate communication between the care staff [23] • Telemedicine systems, which provide immediate feedback to patients [28] • Technology support as home tutorials and social networking
Health organisational system context	• Adequate time to promote new practice [22] • Management incorporation to the implementation process [26] • Motivation and consensus building in organisational culture [19] • To ensure that the staff involved have sufficient training on the intervention [15]
Guidelines context	• Interventions that demonstrated clear and consistent clinical evidence of benefit or good applicability relevant to setting [16] • Guidelines are based on clear and solid recommendations [16]
Health professional context	• Good communication and behaviour change skills of healthcare professionals [15, 28] • Positive attitudes toward innovation and evidence [19]
Patient context	• Structured management plans for patients [15]

#### Political and social context

Frequently mentioned barriers were the absence of a leader that establishes priorities and manages the implementation process [20, 23, 26, 27, 29]; difficulties with teamwork, lack of coordination and disagreement with colleagues [6, 17, 26–29]; the absence of recognising the role of the professional and a lack of clarity of responsibilities [23, 26–28], and financial constrains for the adoption of new interventions [6, 13, 26, 35].

“*The nurses did not know whether to use the clinical practice guidelines or not when the doctors did not agree with each other or with the lead nurses.*” [19]

“*The nurses did not complete the risk assessment because they thought it was the doctor's responsibility*.” [26]

The facilitators were consistent leadership, which creates enthusiasm and provides clear objectives of care [16, 19, 20, 22]; commitment of the multidisciplinary team members and the support of administrators [14, 16, 23]; the exchange of experiences among staff [23]; the superiors’ support for the implementation of the guide [16, 23], and confidence in other experienced colleagues [14, 23].

“*The presence of formal leaders and administrators with positive attitudes and open communication, who believed that clinical practice guidelines could make a difference, was identified as a facilitator.*” [19]

#### Health organisational system context

The most mentioned barriers were the lack of time allowed for researching, studying and implementing the guidelines, and too little time in the medical consultations [14, 19–25, 27–30, 32, 35]. Additional barriers were a shortage of hospital resources and equipment [6, 12, 16–20, 24, 26, 28, 29, 33], and few people in the health area who were dedicated to specialised care [12, 16, 18, 22, 24, 27–30, 33].

“*Time limitations were perceived as barriers for clinicians to start reading the guidelines in depth and in detail.*” [21]

“*The costs associated with educating staff were a limitation*” [26]

Some of the facilitators were the existence of multidisciplinary teams [19, 20, 22]; the application of technology to improve the practice [19, 22, 26], the creation of alarms and automatic reminders to implement the recommendations [14, 22, 26], efficient organisational processes [23, 26], positive attitudes of the health personnel towards change [15, 16], and multi-professional audit meetings, with feedback and good communication [12, 26].

“*Computer applications, such as electronic alerts and reminders, as well as the integration of a risk assessment of venous thromboembolism in the electronic patient record and the admission system.*” [26]

“*Audit meetings are conducted in an informal non-punitive learning environment that provides the opportunity for interaction, discussion and exchange of ideas on changing practice.*” [12]

#### CPG context

The most frequently mentioned barriers were a lack of clarity in the CPG [13, 16–19, 24, 28], a belief that the evidence in the guidelines is incorrect or that the evidence is not sufficient to properly inform [16, 18, 21, 26, 28, 35], a belief that CPGs are too rigid or that they are in conflict with the practice accepted by healthcare personnel and do not always translate to day-to-day tasks [16, 19, 29, 32, 34].

“*The doctors perceived that the guidelines lacked clarity or specificity, indicating that there are problems with the way in which the recommendations are written, for example, the level of detail they provide and whether they are easily implemented in clinical practice.*” [24]

Some of the facilitators within this context were positive perceptions about the usefulness of the CPG [19, 21], guidelines presented in a short and simple format [22], providing brochures for patients [22], recommendations that need minimal resources to be implemented [16, 22], the involvement of end-users in the development, implementation and testing of the guidelines [22], and the use of digital guidelines [22].

“*Nurses believe that CPGs improve the exchange with doctors, decision making, assistance to inexperienced clinicians, reduce medical errors, integrate research into practice and improve the care process.*” [19]

#### Health professional context

The most frequent barriers were the ignorance of the existence of the CPGs or recommendations, the basis of evidence and how to search for information [6, 13, 14, 17–19, 22, 23, 25–28, 32, 33], distrust of one’s ability and fear of implementing the guidelines, fear of harming, damaging the patient-doctor relationship, inexperience and distrust of prescriptions [13, 14, 16, 18, 20, 21, 25, 27, 28, 30, 32, 33], and a lack of familiarity with the guideline recommendations [14–18, 23, 26, 27, 29, 32, 33].

“*Health professionals stated that they were too busy to add this to their practice or simply forgot to complete the requirements for venous thromboembolism care*” [26]

“*Reluctance to prescribe prophylaxis due to the possibility of an adverse reaction or an interaction with another medication that the patient may be taking*” [26]

The most common facilitators were education about the guidelines, specific training or educational programmes [19, 20, 23, 25, 26, 28], high motivation to implement the intervention and a positive attitude [20, 23, 25, 30], and good communication and behavioural change skills of healthcare personnel [16, 20, 28, 30].

“*Nonclinical staff often had a better understanding of process optimization compared to physicians, and different members of the workforce contributed different perspectives and skills to the implementation.*” [16]

#### Patient context

The most frequent barriers in the patient context were the unawareness of patients regarding the guideline, the unawareness about the system characteristics and their disease [13, 15, 20, 28–31, 33], a negative attitude of the patient towards the guide, reluctance to follow the recommendations, and expectations in contrast to the opinion of the doctor [6, 13, 16, 18, 24, 28, 29, 33], a lack of family support [18, 27–31], and inadequate patient–doctor relationships and a lack of confidence on the part of the patient [6, 14, 16, 29, 31, 34].

“*General practitioners are challenged by patient expectations that do not agree with their own views, but still influence the management of the disease*” [24]

“*Doctors and patients had different perceptions of the instructions and as a result, patients sometimes could not understand and apply what their doctor told them to do*” [31]

The most frequently mentioned facilitators in the patient context were good information and early education [20, 24, 25], peer support and feedback between patients [20, 25, 29, 31], interest of the patient or motivation to follow the recommendations [25], and health awareness and an understanding of the consequences of not following the recommendations [29, 31].

“*Four studies described a lack of patient interest in physical activity counselling and in smoking and alcohol counselling as a barrier to using preventive services*.” [25]

## Discussion

This metareview synthesises evidence form 25 systematic reviews that included primary studies form different countries with diverse levels of income and performed within different levels of care. There is consistent evidence showing that absence of a leader or champion of the implementation process within organisations, the lack of time of health professionals, lack of clarity and a lack of credibility in the evidence of the CPG, and the lack of knowledge about the CPG are some of the most frequent barriers.

We found barriers that are not described in the previous comprehensive review developed by Flottorp et al. [6], for example, in the context of CPGs, the belief that CPGs defy clinical judgement and medical autonomy and limit treatment options and a belief that the evidence in the guidelines is incorrect or that the evidence is not sufficient to properly inform decisions. In the patient’s context, new barriers were found such as language and literacy problems, sociocultural beliefs, and personal values that led to a misconception of the disease and difficulty in complying with the recommendations. In the organisational and health system context, there were additional barriers like a lack of education of personnel in subjects such as evidence-based medicine, few people in the health area who were dedicated to specialised care and additional workloads.

In 2008, Francke et al. [5] synthesised systematic reviews to describe which factors affected the implementation of guidelines and provided a view regarding the ‘state of the art’ about the research within this field. They included 12 systematic reviews and found that the characteristics of the guidelines that had a positive influence were as follows: a CPG that was easy to understand, no need for additional specific resources and a CPG that could be easily implemented. The main obstacles for implementation by the professionals were a lack of knowledge, a lack of familiarity, ignorance of the existence of the guidelines and a lack of agreement with the recommendations. Young professionals were more inclined to implement the guidelines; some patients perceived that they did not need guidance or did not accept the recommendations, which was identified in the study as one of the barriers related to patients. The environmental factors that had a negative influence were limited work time, limited human resources, working under pressure and little support from superiors.

The results of the current meta-review have some similarities with the study published by Francke et al. [5], for example, barriers such as a lack of knowledge, a lack of familiarity, ignorance of the existence of guidelines, a lack of agreement with recommendations, limited work time, limited human resources, negative attitudes, working under pressure and little support of superiors. New barriers appear in the political and social context, such as the absence of leadership, difficulties with teamwork, a lack of agreement with colleagues, poor and inopportune communication between professionals and patients, guidelines written in a different language, a lack of linguistic skills, cultural diversity and a lack of attention to poor people and minorities, among others.

One of the limitations mentioned in the study conducted by Francke et al. [5], was the low methodological quality of the systematic reviews that were included in their analysis. In our meta-review, only two reviews were assessed with a low-quality score.

Studies such as those of Baatiema et al. [18] and Craig et al. [23] were conducted in high-income countries, and reported barriers to implementation such as a lack of time and a lack of knowledge and clarity of the guidelines. It is significant to explore barriers and facilitators in low- and middle-income countries, as did the studies by Stokes et al. [12] and Samnani et al. [13]. These reviews included different aspects, such as a lack of access to medicines, deficiencies in transportation and communication systems, the existence of fear and confusion among implementers, policy-makers and government officials, and the inequitable distribution of resources. In the study by Khatib et al. [29], studies conducted in India were included, mentioning disrespect for poverty as one of the barriers of the system. In a study carried out on the Mexican immigrant population, emphasis was placed on the barriers related to problems in caring for minorities and studies conducted with African American patients reported the costs of care and distrust in the care provided as barriers.

### Implications for practice

The results of studies that included low- and middle-income countries can provide information for the development of public policies that improve CPG implementation in those contexts. For example, in Colombia, the lack of attention to minorities, ethnic and cultural differences could represent important barriers given the cultural diversity of the country and the existence of many ethnic groups that have different beliefs, dialects, and views of health and disease.

In general, one of the most mentioned barriers was the short amount of time for the care personnel to study the guidelines, to implement the interventions in the consultation and for the re-evaluation of the patient. The health system has a notable influence on the barriers to implementation, such as the difficulty in accessing services, financial difficulties, a lack of specialised personnel and the additional workload caused by CPGs, which limit implementation.

The socio-political and health organisational barriers could be overcome by strengthening organisational governance arrangements. The working groups in charge of promoting quality of care within healthcare organisations could engage leaders or champions to promote the use of CPGs and they could assign financial and physical resources to facilitate interdisciplinary work, research, and the study and implementation of the CPG. Finally, healthcare organisations could have manuals and protocols for the implementation of CPGs.

In the context of the CPGs, it is necessary to conduct wide dissemination, education and training processes to understand the advantages of evidence-based recommendations. CPGs should have aids, applications, charts, flowcharts and easy ways to find useful information at the time the clinician must make a decision.

To overcome patient barriers, an adequate relationship between professionals and doctors is necessary as well as timely and friendly information so that patients can make decisions that are important for their well-being. There are platforms with information for patients and their families to guide them on how to select the best alternative considering their values and the best available evidence.

Barriers such as limited working time could be overcome increasing the availability of human resources. Other social and cultural barriers, such as lack of attention to poor people and minorities, guidelines written in a different language, and the lack of attention to cultural diversity, could also be overcome if governments include specific strategies within their action plans to address the needs of the vulnerable population.

Regarding the facilitators, the importance of effective communication should be highlighted, both among the working team and in the patient–doctor relationship; if there is good communication, the implementation of the guidelines is facilitated. A committed leader who supports CPG implementation creates a culture of change that favours it. The multidisciplinary work teams play a fundamental role as facilitators and can be a strength when creating implementation strategies and reminders and the use of technology can support implementation. A positive attitude towards change was discussed in several reviews as an important facilitator and is fundamental when implementing strategies.

CPGs that are presented in a clear, simple and short manner are easier to implement, and the end-user is more likely to feel motivated and have a positive attitude towards them. A younger doctor age appears to be a facilitator, which can be explained by the inclusion of the subject of CPGs in the undergraduate programmes and the familiarity of universities with the MBE language.

Patient education and motivation create awareness and facilitate implementation; therefore, strategies should be oriented towards the patient in these two aspects. Technology appears strongly in two systematic reviews, with a reliance on technology such as audio-visual aids, systematised reminders and electronic records. In addition, media campaigns are a strong ally for the dissemination of CPGs and improve their implementation.

Policies should encourage implementation at different levels, motivate managers and administrators to promote education, create well-coordinated multidisciplinary teams with clearly defined roles, facilitate adaptation to change by the professional, and ensure adequate follow-up of patients. Policies should promote integrated care and guarantee access to guidelines and services. Finally, the implementation activities conducted must be adapted to the social, cultural and community context to guarantee their success and sustainability.

### Future research

This meta-review is the starting point of a research project that intends to identify the facilitators and barriers to the implementation of CPGs for the diagnosis and preoperative, intraoperative and postoperative treatment of the amputee, the prescription of the prosthesis and comprehensive rehabilitation, which is a mixed-methods study being conducted in Colombia.

### Limitations

In reviews of reviews, there is always the risk that an included study may appear in different reviews and there will be an overlap of the results. In our review, 2.5% of the primary studies were repeated in the reviews (24/960). Another limitation was that the publication date of the articles included in some reviews was prior to 2006, which could have duplicated the information previously published by Francke et al. [5]. Some factors were identified as a barrier and a facilitator and, in some reviews, it was not explained in what sense that factor was a barrier or a facilitator. This points to the importance of having an agreement on what is defined as a barrier or facilitator.

### Conclusion

The multiple barriers and facilitators described in this systematic meta-review are factors that influence the implementation of evidence in clinical practice. Knowledge of these factors should contribute to the development of a theoretical basis for the creation of CPG implementation strategies in order to improve professional practice and health outcomes for patients.

## Supplementary information

**Additional file 1.**

**Additional file 2.** Search strategies by data sources.

**Additional file 3.** Checklist for Systematic Reviews and Research Syntheses.

## Data Availability

Data generated or analysed during this study are included in this published article and its supplementary information files.

## Abbreviation

CPGs: Clinical practice guidelines
