# Supplementary material for: Individual, health system, and contextual barriers and facilitators for the implementation of clinical practice guidelines: a systematic metareview
Source: Health Res Policy Syst. 2020 Jun 29;18:74. doi: 10.1186/s12961-020-00588-8 (PMC7322919; doi:10.1186/s12961-020-00588-8)
Supplement: Supplementary file 1 — Additional file 1. [file 12961_2020_588_MOESM1_ESM.docx]

**Additional File 1.**

**Individual, Health System, and Contextual Barriers and Facilitators for the Implementation of Clinical Practice Guidelines: A Systematic Meta-Review Protocol**

**Autores**

Verónica Ciro Correa. Médica Fisiatra. Clínica las Americas, Grupo de Rehabilitación en Salud. Universidad de Antioquia, Medellín, Colombia. Email: veronicaciro@gmail.com.

Luz Helena Lugo-Agudelo. Médica Fisiatra, MSc en Epidemiología Clínica. Coordinadora Grupo de Rehabilitación en Salud. Universidad de Antioquia. Clínica las Américas. ORCID: 0000-0002-3467-8835. Email: [luzh.lugo@gmail.com.](mailto:luzh.lugo@gmail.com)

Daniel Camilo Aguirre-Acevedo. Estadístico, PhD en Epidemiología. Grupo de Rehabilitación en Salud, Grupo de Epidemiología Clínica. Universidad de Antioquia, Medellín, Colombia. ORCID: 0000-0002-8195-8821. E[mail: daniel.aguirre@udea.edu.co.](mailto:daniel.aguirre@udea.edu.co)

Jesús Alberto Plata Contreras. Grupo de Rehabilitación en Salud. Departamento de Medicina Física y Rehabilitación, Universidad de Antioquia, Medellín, Colombia. Email: [jesus.plata@udea.edu.co](mailto:jesus.plata@udea.edu.co).

# Ana María Posada Borrero. Médica Fisiatra. Grupo de Rehabilitación en Salud. Universidad de Antioquia, Medellín, Colombia. ORCID: 0000-0002-0727-3269. Ema[il: amposada@gmail.com.](mailto:amposada@gmail.com)

# Daniel Felipe Patiño Lugo. Ingeniero Biomédico, PhD en Políticas en Salud. Grupo de Rehabilitación en Salud, Grupo de Epidemiología Clínica. Universidad de Antioquia, Medellín, Colombia. ORCID: 0000-0003-4030-4255. Email: [gruporehabilitacionsalud@udea.edu.co.](mailto:gruporehabilitacionsalud@udea.edu.co)

Dolly Andrea Castaño Valencia. Estudiante Medicina. Universidad de Antioquia, Medellín, Colombia. Ema[il: andrea-31922@hotmail.com.](mailto:andrea-31922@hotmail.com)

**Abstract**

*Introduction*: Clinical practice guidelines (CPGs) are designed to improve the quality of care and reduce unjustified individual variation in clinical practice. Knowledge of the barriers and facilitators that influence the implementation of the CPG recommendations is the first step in creating strategies to improve health outcomes. The current systematic meta-review will seek to explore the barriers and facilitators for the implementation of the CPG. The objective of the present study is to carry out a systematic meta-review of reviews that explore the barriers and facilitators for the implementation of CPG in the different clinical areas of health and according to social and political, health system / organization, the CPG, the individual and the patient contexts.

*Methods*: For this systematic meta-review, the Cochrane protocol for systematic reviews will be used. A search will be performed in the Pubmed, Embase, Cochrane and Health System Evidence. Systematic reviews (SR) of qualitative, quantitative or mixed-methods studies that identified barriers or facilitators for the implementation of CPGs will be included. Title and abstract selection, full-text evaluation, data extraction, and quality evaluation will be performed by two independent reviewers. To summarize the evidence, we created five contexts to group barriers and facilitators; these contexts are: the political and social, the health organization system, the guide, the health professional and the context of the patient. The evaluation of the methodological quality will be made with “ROBIS: Tool to assess risk of bias in systematic reviews” for the reviews of quantitative studies; and qualitative systematic reviews will be evaluated with the instrument Joanna Briggs Institute (JBI) "Checklist for Systematic Reviews and Research Syntheses"

**Background**

Evidence-based clinical practice guidelines (CPGs) are intended to assist practitioner and patient decisions about appropriate health care for specific clinical circumstances (1).

The CPGs are designed to improve the quality of care and reduce variation in practice through recommendations based on the best available evidence (2). Its true utility is evident when implemented, but sometimes this is complex and unpredictable, creating a large gap between the evidence and daily clinical practice.

Different factors that may influence clinical decisions of the health professional have been described in the literature. Identifying the factors that influence the implementation of the recommendations, that is the barriers and facilitators of the process, is an important first step to achieve those changes and bring effective interventions into practice (3)(4).

Barrier and facilitator studies for the implementation of CPG have shown that the identification of these factors provides information on current clinical practice and can serve as a basis to design better strategies so that their proper use benefits patients, professionals and community in general.

However, the information available in the different types of studies is heterogeneous and multiple primary studies whose objective is to identify the barriers and facilitators for the implementation of different types of CPG, employing qualitative, quantitative or mixed methodologies can be found. Information on barriers and facilitators is important in health practice to achieve the implementation of recommendations with better health outcomes for patients.

In previous evidence synthesis studies, different factors have been described that can influence the clinical decisions of health professionals. In 2008, Francke y cols (5) published a systematic meta-review with a search strategy conducted in 2006. It concluded that the factors that influence the implementation of CPGs could be classified in those related to the characteristics of the guidelines, implementation strategies, professionals, patient, and environment. They included 12 systematic reviews and found that the characteristics of the guidelines that had a positive influence were: the ease of understanding the CPG, having no need for additional specific resources, and whether they could be easily implemented. The main obstacles for the implementation in the professionals were the lack of knowledge, the lack of familiarity, the lack of knowledge of the existence of the guidelines and the lack of agreement with the recommendations. Young professionals were more inclined to implement the guidelines; some patients perceived that they did not need the guideline or did not accept the recommendations, which was identified in the study as one of the barriers related to patients. The environmental factors that negatively influenced are limited work time, limited human resources, work pressure, and little support from superiors. Later, in 2013 Flottorp et al. (6) developed a systematic review, a synthesis of frameworks and an expert consensus process to developed a checklist of factors that prevent or enable improvements on healthcare professionals practice. They found 57 factors and grouped them in those related to the guideline, the individual health professional, the patient, the professional interactions, the incentives and resources, the capacity for organizational change, and the social, political, and legal factors. The increasing literature in this area, in the form of SR and research synthesis may overwhelm health care professionals, manager and other decision-makers seeking to understand how best to implement clinical practice guidelines.

Lau et al. (7) conducted a SR that sought to synthesize the available reviews on the causes of the gap between evidence and practice for the implementation of complex interventions in primary care. Reviews of primary care studies in developed countries were included, while reviews of dental practices and reviews from developing countries were excluded. These authors included different topics: implementation of CPGs, quality of care and treatment of the disease, implementation of technology-based interventions, and public health and prevention programs. Only the subtopics of CPG implementation and health care management included topics relevant to the implementation of MBE, such as the chronic disease care model, advanced care planning in palliative care, and nursing-led care. The current meta-review, unlike the study published by Lau et al., included all levels of care, both in developed and developing countries.

The objective of this systematic review is to synthesize and update the information published from December 2006 to February 2017 and then it was update between February 2017 until January 10, 2018 from systematic reviews that identify barriers and facilitators for the implementation of clinical practice guidelines at all levels of care and in different health areas.

**Objective**

The objective of the current study was to carry out a systematic meta-review of reviews that explore the barriers and facilitators for the implementation of CPGs in the different clinical areas of health and according to the social political contexts, the health/organizational system, the CPG itself, the individual and the patient, from 2006 to January 10, 2018.

**Methods**

The structure of the review process was adapted according to the guidelines of the Cochrane Collaboration (8)

**Criteria for considering studies for this review**

*Types of studies*

Systematic reviews or review of reviews that identify barriers and facilitators for the implementation of the CPGs.

Systematic reviews or review of reviews that include qualitative, quantitative, or mixed-method studies, and that contain the description of the search and quality assessment of the included studies.

Studies published in the period January 2006 to November 30, 2017

Target population: patients, health service providers and professionals.

Studies published in any language.

*Types of outcome measures*

Primary: Barriers and facilitators according to the social political contexts, the health/organizational system, the CPG itself, the individual and the patient.

Secondary: Barriers and facilitators identified by health area, target population (patients, health service providers and professionals), country of research and level of care.

Exclusion criteria:

Guidelines studies based on expert consensus or protocols.

Guidelines-based studies without systematic analysis of scientific literature (not evidence-based).

Primary studies (controlled, observational, narrative, or mixed-method clinical trials)

Studies whose objective was not to evaluate barriers and facilitators

**Search methods for study identification**

*Electronic searches*

A search will be performed from December 2006 to January 10, 2018.The meta-review published by Francke et al. (5) included studies that were published until November 2006; therefore, a search was conducted from December 2006 in Pubmed, Embase, Cochrane, Health System Evidence databases and International Guideline Library (G-I-N).

Reviews in all languages.

**Pubmed:**

((Guideline[MeSH] OR Guideline Adherence[MeSH] OR Practice Guidelines as Topic[MeSH] OR Guidelines as Topic[MeSH] OR guideline*[tiab] OR recommendation*[tiab] OR consensus[tiab]) AND (barrier*[tiab] OR obstacle*[tiab] OR impediment*[tiab]) AND (implement*[tiab] OR complian*[tiab] OR adherence[tiab] OR application[tiab] OR adoption[tiab])) AND systematic[sb]

**Embase:**

('practice guideline'/exp/mj or 'protocol compliance'/exp/mj or 'patient compliance'/exp/mj or 'guideline':ab,ti) and (('barrier*':ab,ti or 'obstacle*':ab,ti or 'impediment*':ab,ti) and ('implement*':ab,ti or 'complian*':ab,ti or 'adherence*':ab,ti or 'application':ab,ti or 'adoption':ab,ti)) and ([cochrane review]/lim or [systematic review]/lim or [meta analysis]/lim or [controlled clinical trial]/lim or [randomized controlled trial]/lim)

**Cochrane:**

guideline:ti,ab,kw

#2 barrier:ti,ab,kw or obstacle:ti,ab,kw or impediment:ti,ab,kw

#3 (#1 AND #2)

**Health System Evidence**

Barriers or facilitators

**Data extraction and analysis**

After removing duplicates, two independent researchers will review titles and abstracts according to established selection criteria, and disagreements will be resolved by a third reviewer. Then two independent reviewers will conduct a full-text review and a third reviewer will resolve disagreements.

***Assessment of risk of bias in included studies***

Quantitative studies

The evaluation of the methodological quality will be made with “ROBIS: Tool to assess risk of bias in systematic reviews” for the reviews of quantitative studies (9). Two reviewers will apply the methodological quality evaluation criteria independently, any disagreement will be reviewed with a third evaluator.

Qualitative studies

The quality evaluation of the reviews was conducted independently by two reviewers with the tool developed by the Joanna Briggs Institute (JBI) "Checklist for Systematic Reviews and Research Syntheses" (10)

***Data extraction and management***

Data extraction will be using forms designed by the researchers with the following information: country where the individual studies included in each review were conducted, income level of those countries, level of care (that is, primary, secondary or tertiary care), health topic, type of review (quantitative, qualitative or mixed depending on the type of studies included in the review) and the results and conclusions synthesized by the review authors.

***Data Synthesis***

The qualitative synthesis will be carried out in three stages (14). The first stage will seek to determine how the studies were related, taking into account the political/social context, the health / organization system, the CPG, individuals and patients, according to the model of Flottorp et al (6). Key concepts will be extracted from the results and discussions of the included reviews and related to the contexts or themes described. The second stage will compare the studies with the key themes or concepts identified in the first stage; the data will be grouped into similar concepts. An array will be created with the revisions in the columns and the contexts in rows, allowing you to explore the relationships between them. Finally, the third stage will include the synthesis of the problems identified in the previous stages, and a final analysis.

**Figure 1- Prisma**


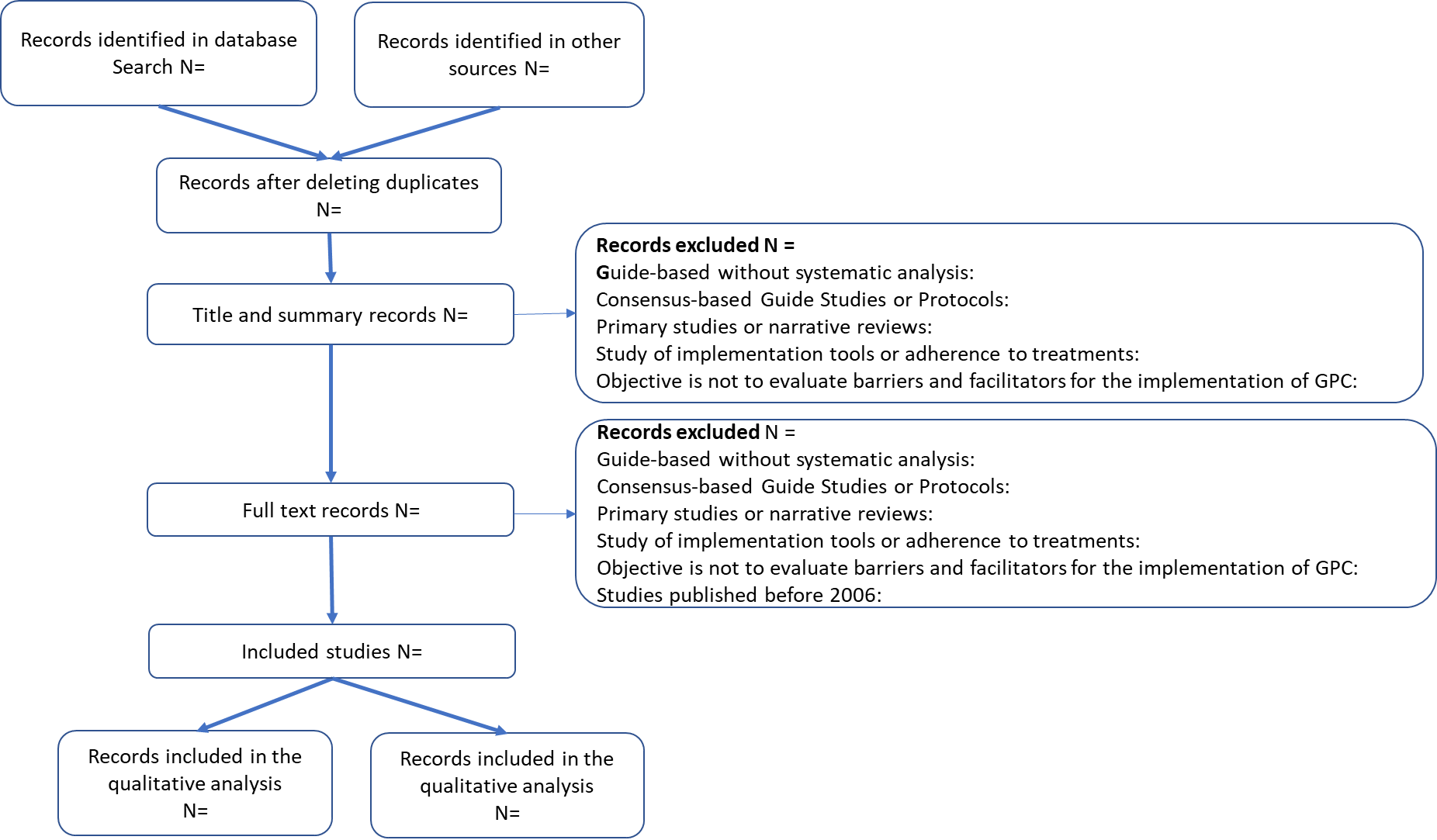


**Bibliography**

1. Bhandari M, Giannoudis P V. Evidence-based medicine: What it is and what it is not. Injury. 2006;37(4):302–6.

2. Greenfield S, Steinberg E. Sumary. In: Clinical Practice Guidelines We Can Trust. National Academies Press; 2011. p. 4.

3. Grol R, Grimshaw J. From best evidence to best practice: Effective implementation of change inpatients’ care. Lancet. 2003;362(9391):1225–1230.

4. Rabin BA, Brownson RC, Haire-Joshu D, Kreuter MW, Weaver NL. A glossary for dissemination and implementation research in health. J Public Heal Manag Pract. 2008;14(2):117–23.

5. Francke AL, Smit MC, de Veer AJ, Mistiaen P. Factors influencing the implementation of clinical guidelines for health care professionals: a systematic meta-review. BMC Med Inform Decis Mak. 2008;8(38):1–11.

6. Flottorp SA, Oxman AD, Krause J, Musila NR, Wensing M, Godycki-Cwirko M, et al. A checklist for identifying determinants of practice: A systematic review and synthesis of frameworks and taxonomies of factors that prevent or enable improvements in healthcare professional practice. Implement Sci [Internet]. 2013;8(35):1–11. Available from: http://www.implementationscience.com/content/8/1/35

7. Lau R, Stevenson F, Ong BN, Dziedzic K, Treweek S, Eldridge S, et al. Achieving change in primary care — causes of the evidence to practice gap : systematic reviews of reviews. Implement Sci [Internet]. 2016;11(40):1–39. Available from: http://dx.doi.org/10.1186/s13012-016-0396-4

8. Centro Cochrane Iberoamericano T. Manual Cochrane de Revisiones Sistemáticas de Intervenciones, versión 5.1. 0 [Internet]. Barcelona: Centro Cochrane Iberoamericano; 2012. 1-639 p. Available from: http://www.cochrane.es/?q=es/node/269%0A4

9. Higgins JPT, Caldwell DM, Whiting P, Savovi J. ROBIS : A new tool to assess risk of bias in systematic reviews was developed. 2016;69:225–34.

10. The Joanna Briggs Institute. Checklist for Systematic Reviews and Research Syntheses [Internet]. The Joanna Briggs Institute. 2016. Available from: http://joannabriggs.org/research/critical-appraisal-tools.html www.joannabriggs.org
