# Supplementary material for: Individual, health system, and contextual barriers and facilitators for the implementation of clinical practice guidelines: a systematic metareview
Source: Health Res Policy Syst. 2020 Jun 29;18:74. doi: 10.1186/s12961-020-00588-8 (PMC7322919; doi:10.1186/s12961-020-00588-8)
Supplement: Supplementary file 2 — Additional file 2. Search strategies by data sources. [file 12961_2020_588_MOESM2_ESM.docx]

**Additional file 2- Search strategies by data sources**

Date. 1. A search was conducted from December 2006 to February 2017 and then it was update between February 2017 until January 10, 2018 in Pubmed, Embase, Cochrane, International Guideline Library (G-I-N) and Health System Evidence databases with the terms "Guidelines", "barriers" and "implement*", with their respective medical subject heading (MeSH) and synonyms, applying the subset to select systematic reviews, meta-analyses and guidelines.

| Data sources | Strategy | Results |
| --- | --- | --- |
| Pubmed | ((Guideline[MeSH] OR Guideline Adherence[MeSH] OR Practice Guidelines as Topic[MeSH] OR Guidelines as Topic[MeSH] OR guideline*[tiab] OR recommendation*[tiab]) AND (barrier*[tiab] OR obstacle*[tiab] OR impediment*[tiab]) AND (implement*[tiab] OR complian*[tiab] OR adherence[tiab] OR application[tiab] OR adoption[tiab])) AND systematic[sb] | 1103 |
| Embase | ('practice guideline'/exp/mj or 'protocol compliance'/exp/mj or 'patient compliance'/exp/mj or 'guideline':ab,ti) and (('barrier*':ab,ti or 'obstacle*':ab,ti or 'impediment*':ab,ti) and ('implement*':ab,ti or 'complian*':ab,ti or 'adherence*':ab,ti or 'application':ab,ti or 'adoption':ab,ti)) and ([cochrane review]/lim or [systematic review]/lim or [meta analysis]/lim or [controlled clinical trial]/lim or [randomized controlled trial]/lim) | 363 |
| Cochrane | #1 guideline:ti,ab,kw  #2 barrier:ti,ab,kw or obstacle:ti,ab,kw or impediment:ti,ab,kw  #3 (#1 AND #2) | 58 |
| Health system evidence | Barrier AND facilitator | 156 |
| International Guideline Library | Guidelines / Barriers | 3 |

The following controlled and free terms were included in the strategy to guarantee the scope and relevance of the searches.

**Relevant MeSH terms:**

"guidelines as topic"[MeSH Terms]

"guideline adherence"[MeSH Terms]

"practice guidelines as topic"[MeSH Terms]

"guidelines as topic"[MeSH Terms]

**Free terms (related synonyms and antonyms):**

"guideline*"[Title/Abstract]

"recommendation*"[Title/Abstract]

"barrier*"[Title/Abstract]

"obstacle*"[Title/Abstract]

"impediment*"[Title/Abstract]

"implement*"[Title/Abstract]

"complian*"[Title/Abstract]

"adherence"[Title/Abstract]

"application"[Title/Abstract]

"adoption"[Title/Abstract]

Each term to be included was independently tested to ensure the necessary redundancy in the strategy. (The results presented have the addition of the time elapsed since 2018 when the searches for the synthesis were performed)

| **#** | **Query** | **Search Details** | **Results** |
| --- | --- | --- | --- |
| 17 | #9 OR #10 OR #11 OR #12 OR #13 OR #14 OR #15 OR #16 | "barrier*"[Title/Abstract] OR "obstacle*"[Title/Abstract] OR "impediment*"[Title/Abstract] OR "implement*"[Title/Abstract] OR "complian*"[Title/Abstract] OR "adherence"[Title/Abstract] OR "application"[Title/Abstract] OR "adoption"[Title/Abstract] | 1,770,190 |
| 16 | adoption[tiab] | "adoption"[Title/Abstract] | 49,258 |
| 15 | application[tiab] | "application"[Title/Abstract] | 787,536 |
| 14 | adherence[tiab] | "adherence"[Title/Abstract] | 118,185 |
| 13 | complian*[tiab] | "complian*"[Title/Abstract] | 129,542 |
| 12 | implement*[tiab] | "implement*"[Title/Abstract] | 485,022 |
| 11 | impediment*[tiab] | "impediment*"[Title/Abstract] | 8,546 |
| 10 | obstacle*[tiab] | "obstacle*"[Title/Abstract] | 47,775 |
| 9 | barrier*[tiab] | "barrier*"[Title/Abstract] | 294,063 |
| 7 | #1 OR #2 OR #3 OR #4 OR #5 OR #6 | "guidelines as topic"[MeSH Terms] OR "guideline adherence"[MeSH Terms] OR "practice guidelines as topic"[MeSH Terms] OR "guidelines as topic"[MeSH Terms] OR "guideline*"[Title/Abstract] OR "recommendation*"[Title/Abstract] | 639,611 |
| 6 | recommendation*[tiab] | "recommendation*"[Title/Abstract] | 255,873 |
| 5 | guideline*[tiab] | "guideline*"[Title/Abstract] | 349,092 |
| 4 | Guidelines as Topic[MeSH] | "guidelines as topic"[MeSH Terms] | 159,954 |
| 3 | Practice Guidelines as Topic[MeSH] | "practice guidelines as topic"[MeSH Terms] | 117,259 |
| 2 | Guideline Adherence[MeSH] | "guideline adherence"[MeSH Terms] | 31,891 |
| 1 | Guideline[MeSH] | "guidelines as topic"[MeSH Terms] | 159,954 |
